# Parenchyma-Sparing Hepatectomy with Vascular Reconstruction Techniques for Resection of Colorectal Liver Metastases with Major Vascular Invasion

**DOI:** 10.1245/s10434-016-5378-x

**Published:** 2016-07-11

**Authors:** Saiho Ko, Yuuki Kirihataya, Masanori Matsusaka, Tomohide Mukogawa, Hirofumi Ishikawa, Akihiko Watanabe

**Affiliations:** Department of Surgery, Nara Prefecture General Medical Center, Nara, Japan

## Abstract

**Background:**

Resectability of colorectal liver metastasis (CRLM) depends on major vascular involvement and is affected by chemotherapy-induced liver injury. Parenchyma-sparing with combined resection and reconstruction of involved vessels may expand the indications and safety of hepatectomy.

**Methods:**

Of 92 patients who underwent hepatectomy for CRLM, 15 underwent major vascular resection and reconstruction. The reconstructed vessels were the portal vein (PV) in five cases, the major hepatic vein (HV) in nine cases, and the inferior vena cava in six cases.

**Results:**

All PV reconstructions were direct anastomoses. The HV was reconstructed with an autologous inferior mesenteric venous patch or an external iliac vein interposition graft. Total hepatic vascular exclusion was performed for six patients. Of nine patients with HV reconstruction, three had tumors involving all three major HVs, in whom the left HV was reconstructed as an only vein after extended right hepatectomy. In another six patients, multiple bilobar tumors or tumors in the liver that had chemotherapy-induced injury involved one or two HVs. Parenchyma-sparing by reconstruction of the HV was performed to secure the residual liver function. The patients with vascular reconstruction had an operative time of 462 ± 111 min and a blood loss of 1278 ± 528 mL. No complication classified as Clavien–Dindo 3 or more developed. The median hospital stay was 17 days (range 8–26 days). The cumulative 5-year survival rate for all the patients was 54.6 %, with no significant difference according to vascular reconstruction.

**Conclusion:**

Parenchyma-sparing hepatectomy combined with vascular reconstruction is a useful option to avoid major hepatectomy among various procedures for resection of CRLM with major vascular invasion.

Despite recent advances in chemotherapy, surgical resection remains the only treatment that can ensure long-term survival for patients with colorectal liver metastases (CRLM). Major vascular involvement is one of the most common reasons for unresectability. Although a free surgical margin is of special importance for long-term survival,^1^–^5^ major vascular involvement often is a barrier against curability.

Hepatectomy for CRLM with massive involvement of portal vein (PV) bifurcation, multiple major hepatic veins (HVs), or the inferior vena cava (IVC) still is challenging. Tumors involving all three HVs have been considered unresectable or even a contraindication for hepatectomy.^6^ Reconstruction of a single vein of residual liver is required after major hepatectomy, with combined resection of the three HVs.^7^,^8^

Multiple bilobar tumors are another difficult situation.^5^,^9^,^10^ When one of the tumors involves a major HV, major hepatectomy usually is considered. However, the volume of the residual liver can be insufficient due to the significant amount of hepatectomy for tumors in the contralateral liver. Even if the tumor is solitary, it is better to avoid major hepatectomy when the background liver has been injured with chemotherapy-induced damage. In these situations, parenchyma-sparing hepatectomy with combined resection and subsequent reconstruction of involved vessels may be an alternative to major hepatectomy.

Sporadic reports describe hepatectomy with major vascular reconstruction for CRLM.^7^,^8^,^11^–^16^ Nevertheless, the number of patients and the variation of procedures in the respective reports are limited. Technical aspects, safety, and the impact on long-term survival are still to be elucidated. Moreover, the concept of parenchyma-sparing hepatectomy is uncommon in these studies.

The current study aimed to show the specific role of parenchyma-sparing hepatectomy in various procedures of combined vascular reconstruction for resection of CRLM with major vascular involvement.

## Patients and Methods

### Patients

From 2008 to 2014, 313 patients underwent hepatectomy at our hospital. Of these patients, 92 underwent hepatectomy for CRLM, including 15 patients with major vascular resection and reconstruction. These 15 patients did not include patients with simple wedge resection, performed with side-clamping and suturing. The 15 patients included 2 patients who underwent extensive wedge resection of the IVC that required total hepatic vascular exclusion (THVE). Written informed consent for the use of clinical data for research works in an anonymous setting was obtained from every patient. The institutional review board of Nara Prefecture Medical Center Hospital approved this clinical study.

### Indication Criteria for Hepatectomy and Vascular Reconstruction

Tumor status was evaluated by using three-phase contrast-enhanced computed tomography (CT). Contrast-enhanced magnetic resonance imaging (MRI) was added when needed. The indication criteria for surgical resection of CRLM required that there be no severe comorbid systemic condition, no uncontrollable extrahepatic metastases, curative intent possible for all liver metastases, and a functional liver remnant exceeding 30 % of the whole liver. The requirement was affected by the severity of chemotherapy-induced liver injury, indicated by a blood chemistry test or the indocyanine green retention rate at 15 min.

### Surgical Procedures

The abdomen was opened via a J-shape incision. The relationship between the tumors and major intrahepatic vasculatures were confirmed by intraoperative ultrasonography. The liver was mobilized as broad as required for the planned surgery. The hepatic parenchyma was transected using the clamp-crushing method. Thin vessel branches were burned by electrocautery. The thicker branches were ligated and divided. The intermittent Pringle’s maneuver was applied routinely, involving a 15-min period for clamping and a 5-min period for release. For vascular reconstruction, continuous suture was performed principally with 6-0 or 5-0 prolene. No antithrombotic agent was administered after surgery regardless of the procedures.

### Evaluation of Operative Morbidity and Mortality

The severity of postoperative complications was classified according to Clavien–Dindo criteria.^17^ Hepatic failure was defined as a serum total bilirubin level higher than 5 mg/dL after postoperative day 5 or later. Operative mortality was defined as all in-hospital deaths and deaths within 90 days after surgery.

### Follow-up Schedule and Adjuvant Chemotherapy

The patients received CT or MRI evaluation every 4–6 months after discharge. A blood test and physical examination were applied every 1–6 months until 5 years after the last intervention. Of the 15 patients, 9 received adjuvant chemotherapy after hepatectomy.

### Statistical Analysis

The values are shown as mean ± standard deviation or as median with minimum and maximum values in parenthesis. Survival rates for the patients were calculated by the Kaplan–Meier method and compared by the log-rank test. A *p* value lower than 0.05 was considered statistically significant.

## Results

The 15 patients who underwent hepatectomy with major vascular reconstruction included 9 men and 6 women with a median age of 69 years (range 36–78 years). The CRLM was synchronous in seven patients and metachronous in eight patients. The reconstructed vessels were PV in five patients, HV in nine patients, and IVC in six patients, whereas two or three types of vessels were reconstructed at the same time in some patients (Table 1).

**Table 1:** Surgical procedures

Case no.	Category of vessels reconstructed	No. of tumors	Maximum tumor size (cm)	Extent of hepatectomy	Reconstructed vessels	Reconstruction procedures and graft	THVE (min duration)
1	PV	2	7.0	Right hepatectomy	Left PV	Direct anastomosis	No
2	PV	6	16.0	Right hepatectomy	Left PV	Direct anastomosis	No
3	PV	1	14.3	Right hepatectomy	Left PV	Direct anastomosis	No
4	PV	2	10.0	Right hepatectomy	Left PV	Direct anastomosis	No
5	PV, HV, IVC	2	9.0	Extended right hepatectomy	Left PV LHV	IMV patch for HV Direct anastomosis for PV	Yes (23)
6	HV, IVC	1	20.0	Extended right hepatectomy	LHV IVC	IMV patch for HV	Yes (9)
7	HV	8	8.0	Extended right hepatectomy: partial × 1	LHV	Anastomosis with external iliac vein interposition	No
8	HV	4	2.5	Partial × 4	RHV	IMV patch graft	No
9	HV, IVC	15	5.5	Extended anterior sectionectomy: partial × 13	IVC RHV	Direct	Yes (7)
10	HV, IVC	9	5.5	Segment 8: partial × 5	RHV, LHV	Direct	Yes (10)
11	HV	7	5.7	Central bisectionectomy: partial × 2	RHV	IMV patch graft	No
12	HV	6	3.5	Partial in segments 58 and 7, segment 1	LHV	IMV patch graft	No
13	HV	2	3.0	Partial × 2	MHV	IMV patch graft	No
14	IVC	10	18.0	Right hepatectomy, segment 3: partial × 2	IVC	Direct	Yes (7)
15	IVC	1	6.0	Right hepatectomy	IVC	IMV patch	Yes (26)

THVE, total hepatic vascular exclusion; PV, portal vein; HV, hepatic vein; IVC, interior vena cava; LHV, left hepatic vein; IMV, inferior mesenteric vein; RHV, right hepatic vein; MHV, middle hepatic vein

### Procedures of Vascular Reconstruction (Table 1)

*PV Reconstruction* (*Cases* 1–5 *in Table* 1) The tumors were exclusively large (7–16 cm), involving portal bifurcation. Right hepatectomy and direct end-to-end anastomosis between the portal trunk and the left PV branch was performed for all the patients. The most important issue during this procedure was to judge the necessity of bile duct resection and subsequent bilioenteric anastomosis. The relation between the cutting line of the bile duct and the point of biliary bifurcation was confirmed by intraoperative cholangiography in all the patients. Although no patients required bilioenteric anastomosis in this series, transient external biliary drainage was provided for two patients by placement of a C-tube to avoid stenosis of the sutured site of the biliary stump.

**Fig. 1:**
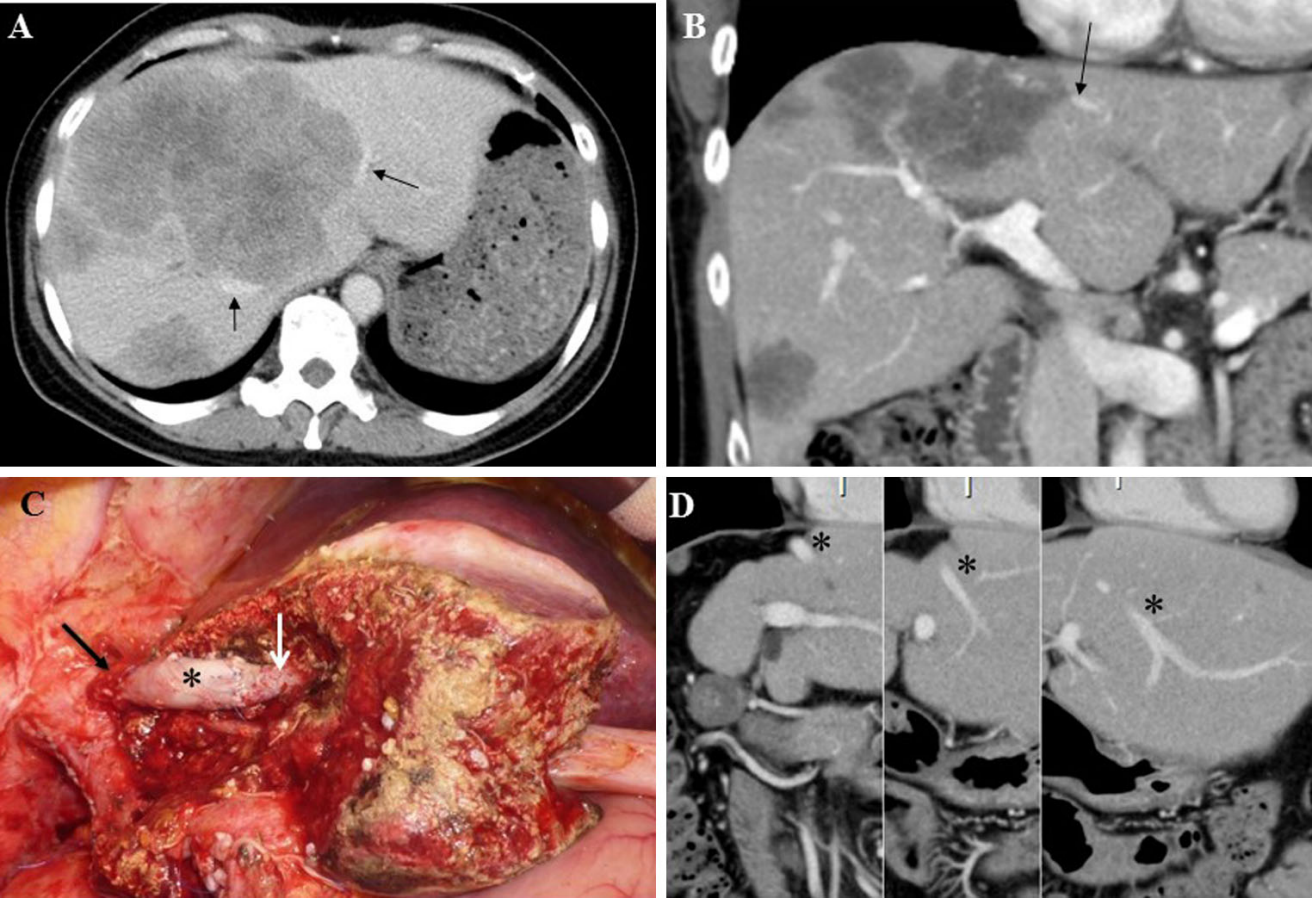
Extended right hepatectomy with left hepatic vein (LHV) reconstruction using interposition of an autologous external iliac vein graft (case 7 in Table 1). Initially, the patient’s tumor condition was diagnosed as unresectable because of massive multiple tumors predominantly in the right liver, with invasion of the right hepatic vein (RHV) and the trunk of the middle hepatic vein (MHV) and LHV (*arrows* on **a**). After seven courses of mFOLFOX6/panitumumab, the tumors shrank significantly, and the trunk of the MHV + LHV still was involved by the tumor (*arrow* on **b**). An external iliac vein interposition graft (*asterisk*) 5 cm long has been anastomosed between the distal stump of the LHV (*white arrow*) and the interior vena cava (IVC) orifice of the MHV + LHV trunk (*black arrow* on **c**). A computed tomography (CT) scan 12 months after hepatectomy shows the reconstructed LHV to be patent (*asterisk* on **d**). At this writing, the patient is alive without recurrence 24 months after the hepatectomy

*Reconstruction of an Only Vein of the Liver Remnant* (*Cases* 5–7 *in Table* 1) Three of nine patients with HV reconstruction (cases 5–13), had tumors involving all three major HVs (cases 5–7). Because large dominant tumors existed in the right liver (diameter 8–20 cm), the left hepatic vein (LHV) was reconstructed as a single vein of the liver remnant after extended right hepatectomy with the middle hepatic vein (MHV). The crafted autologous inferior mesenteric vein (IMV) was used as a patch graft in two of three patients (cases 5 and 6). In the remaining patient (case 7, Fig. 1), the autologous external iliac vein graft was used as an interposition graft and anastomosed between the proximal stump of the LHV + MHV trunk and the distal stump of the LHV as an only drainage route of the residual liver. In two patients, THVE was applied because the HV invasion reached its orifice on the IVC.

**Fig. 2:**
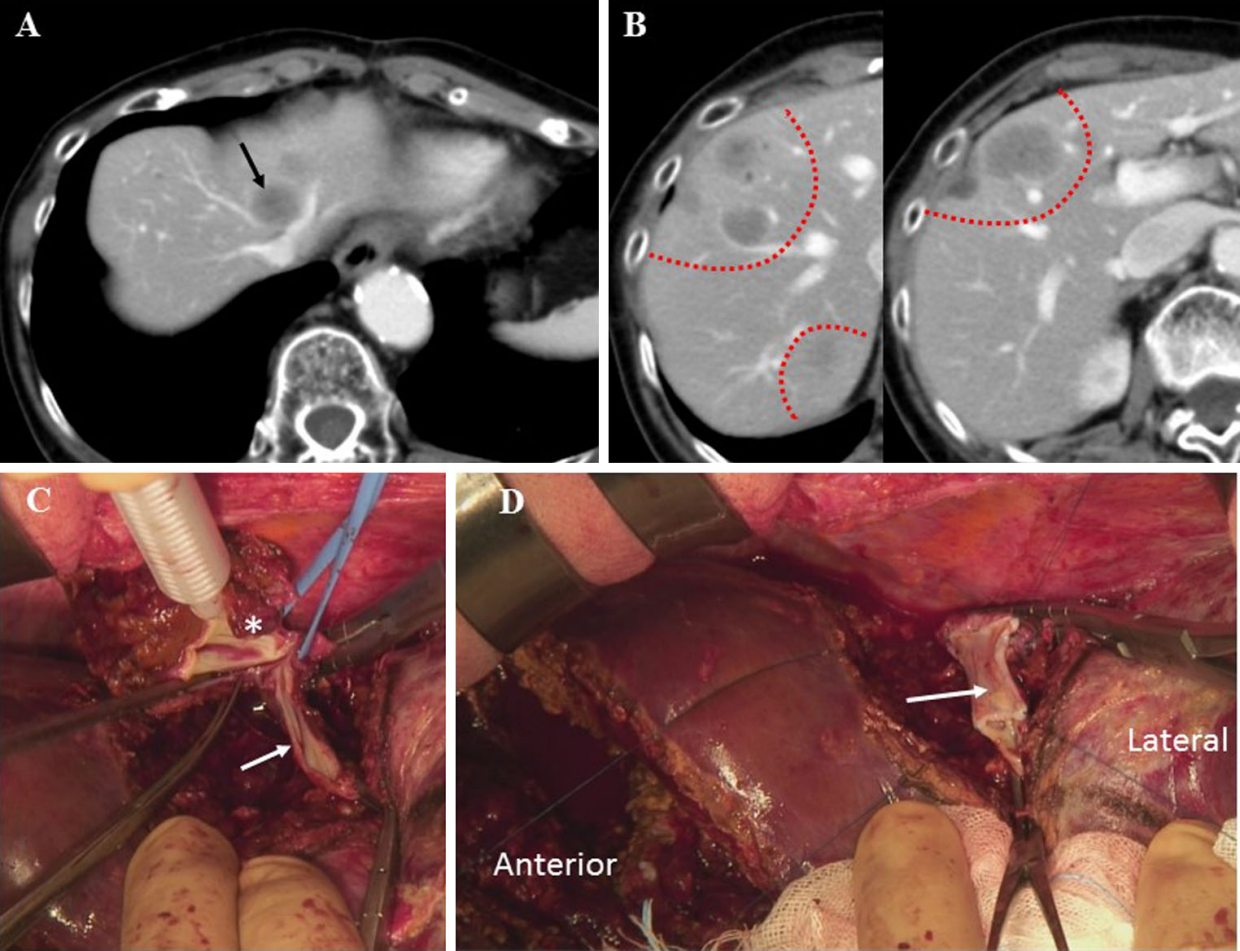
Partial resection of segments 4 and 8 associated with a large amount of hepatectomy of the anterior section and segment 7 (case 12 in Table 1). The ventral wall of the middle hepatic vein (MHV) and the left hepatic vein (LHV) is involved by the tumor occupying segments 4 and 8 (*arrow* on **a**). A significant amount of the anterior section and segment 7 must be resected for other tumors (*red dotted lines*) (**b**). The tumor in segments 4 and 8 was resected with the whole MHV and the anterior wall of the LHV (*asterisk* on **c**). The *white arrow* shows the remaining posterior wall of the LHV (**c**). The defect of LHV was reconstructed with an inferior mesenteric vein (IMV) patch graft stretched between the proximal orifice of the trunk of MHV + LHV and the distal orifice of the LHV, with four points-stay stitches (**d**). “Anterior” shows the large defect of the right anterior section (**d**). “Lateral” shows the left lateral section spared by LHV reconstruction (**d**)

*Parenchyma-Sparing by Reconstruction of the HV* (*Cases* 8*–*13 *in Table* 1) The HV was reconstructed for the purpose of parenchyma-sparing in these patients instead of major hepatectomy. The tumors involved one or two of the three major HVs. Major hepatectomy, including involved HVs, might be a usual choice in such a situation. However, most of these patients had multiple bilobar tumors. Because both a major hepatectomy and a significant amount of partial hepatectomy of the contralateral liver would have resulted in an insufficient liver remnant, parenchyma-sparing by reconstruction of the HV was performed to avoid major hepatectomy (Fig. 2). Even if the tumor is unilobar, major hepatectomy should be avoided when the background liver has been injured by chemotherapy before surgery (case 13). For two patients in whom involvement of the HV extended to the IVC, THVE was applied. The HV was reconstructed by IMV patch grafting in four patients, and repaired by suturing in the remaining two patients under THVE.

*IVC Reconstruction Under THVE* (*Cases* 14 *and* 15 *in Table* 1) The procedure was applied for the tumor involving the IVC extensively around the right hepatic vein (RHV). Although the invasion around the RHV usually can be repaired by simple suture with side-clamping of the IVC, the involvement of IVC was so extensive leftward, it was not possible to apply side-clamping of the IVC in these patients. Consequently, THVE was needed.

### Patency of the Reconstructed Vessels

The patency of the reconstructed vessels was confirmed by Doppler ultrasonography during the first postoperative week in all the patients. One patient with LHV reconstruction using an IMV patch graft as a parenchyma-sparing procedure without major hepatectomy showed obstruction of the reconstructed vein without congestion or ischemia of the liver shown on the CT image 4 months after surgery. Others showed patent reconstructed vessels on the follow-up CT.

### Operative Parameters, Postoperative Course, and Long-Term Survival

**Fig. 3:**
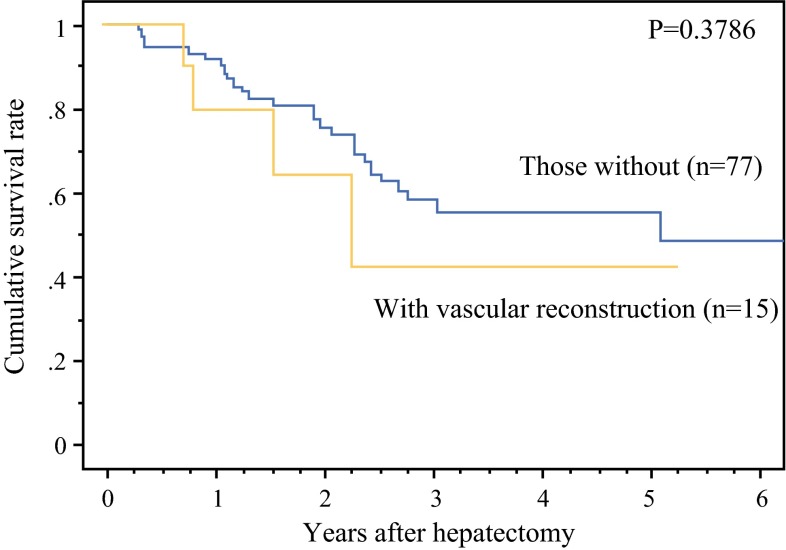
Cumulative overall survival rate after hepatectomy for colorectal liver metastasis according to major vascular reconstruction. The survival rate for the 15 patients who underwent hepatectomy with major vascular reconstruction was compared with that of 77 patients who did not in the same era. The two groups did not show a statistically significant difference

For 15 patients with vascular reconstruction, the operative time was 462 ± 111 min, and the blood loss was 1278 ± 528 mL (Table 2). No complication related to vascular reconstruction was observed. No complication classified as Clavien–Dindo 3 or higher and no operative mortality occurred. The median hospital stay was 17 days (range 8–26 days), and all the patients were discharged within 1 month. The cumulative 5-year survival rate for all 92 patients was 54.6 %. The survival of the patients with and without major vascular reconstruction did not differ significantly (Fig. 3).

**Table 2:** Operative parameters and postoperative course of 15 patients with major vascular reconstruction

Parameters	Values or no. of patients
Mean operation time (min)	462 ± 111
Mean blood loss (mL)	1278 ± 528
Complications: Calvien–Dindo classification	
1	4
2	3
3–5	0
Operative mortality	0
Median hospital stay: days (range)	17 (8–26)

## Discussion

The current study showed short- and long-term results of hepatectomy in combination with major vascular resection and reconstruction for CRLM. The analysis showed some patterns of procedure. To save patients, PV reconstruction for hepatic hilar invasion and HV reconstruction of the only vein after major hepatectomy for involvement of all of three major HVs were indispensable methods. Parenchyma-sparing by reconstruction of the HV instead of major hepatectomy was indicated for multiple bilobar metastases and chemotherapy-induced liver injury. Although the procedure was sometimes complex, the operative data and morbidity were acceptable. The long-term survival rate also was satisfactory compared with the rates in other studies.^18^–^20^

The current study classified the patterns of combined major vascular resection/reconstruction procedures into four types (see “*Procedures of Vascular Reconstruction*” section), including parenchyma-sparing hepatectomy. Such classification of technical variations has not been reported in other literatures.

For the patients with large tumors involving the hepatic hilum, PV reconstruction was indicated exclusively with right hepatectomy. The reason may have been that the right portal branch was shorter than the left portal branch. The most important point during this procedure was to set an accurate cutting line of the bile duct. Although we have not experienced a case that needs bile duct resection and bilioenteric anastomosis, it should be considered that the patient may become vulnerable to reflux cholangitis after bilioenteric anastomosis. This could be important for CRLM patients who may receive systemic chemotherapy for adjuvant therapy or recurrence.

When the tumors involve all three major hepatic veins, HV reconstruction is an only way to indicate curative hepatectomy. It may be the ultimate parenchyma-sparing for the last liver remnant. The reports of hepatectomy with HV reconstruction show only a limited number of patients who underwent reconstruction of an only vein of the residual liver.^7^,^8^,^11^,^14^,^21^ We experienced three cases of reconstruction of the LHV as the only vein of the liver remnant after extended right hepatectomy including RHV, MHV, and LHV. This procedure was psychologically challenging because the ischemic time of the liver had to be shortened, and failure of the reconstruction would be lethal. Careful planning, fine technique of hepatectomy, and accurate vascular anastomosis were required for success.

Parenchyma-sparing with HV reconstruction can be an alternative to major hepatectomy. The procedure was indicated mainly for patients with multiple bilobar tumors. Because nonanatomic hepatectomy does not have a negative impact on the prognosis for patients with CRLM,^2^–^5^ parenchyma-sparing with this procedure may be a useful option. One report described HV reconstruction in a nonmandatory setting for patients with multiple bilobar CRLM based on a concept similar to ours.^14^

Recently, conversion therapy has been an useful strategy for initially unresectable CRLM patients, and many patients have received intensive chemotherapy before hepatectomy.^22^,^23^ Hepatectomy for patients with chemotherapy-induced liver injury is prone to postoperative morbidity and liver failure.^24^ In such situations, parenchyma-sparing hepatectomy with HV reconstruction may be a choice to avoid major hepatectomy. Of course, preoperative portal vein embolization (PVE) with subsequent major hepatectomy is a simpler approach. However, PVE induces humoral growth factors that can stimulate proliferation of colorectal cancer cells and promote recurrence of CRLM after hepatectomy.^25^,^26^ Although two-stage hepatectomy and ALPPS (associating liver partition and portal vein ligation for staged hepatectomy) also may be possible solutions, the feasibility and safety of these approaches are subjects of an ongoing debate due to the high rate of treatment failure and interstage morbidity.^9^,^10^ Nevertheless, these methods include major hepatectomy. If intrahepatic recurrence develops after major hepatectomy, the methods of hepatectomy are restricted. The advantage of parenchyma-sparing with HV reconstruction may be preservation of major vasculatures, which reserves variation of the hepatectomy procedure in case of future intrahepatic recurrence. This point is worth considering.

The techniques used for parenchyma-sparing hepatectomy are similar to those reported previously.^11^,^12^,^14^–^16^ Azoulay et al.^15^ reported surgical results for 84 consecutive patients who had 97 vascular reconstruction combined with liver resection. This is the largest series with this approach. However, no report has described parenchyma-sparing with vascular reconstruction to avoid major hepatectomy. The concept results from recent refinement and stability of vascular reconstruction techniques. The current study showed that parenchyma-sparing by vascular reconstruction can be a dominant choice rather than major hepatectomy of the involved major vessel side.

The THVE procedure was applied for 6 of 15 patients without veno-venous bypass because the duration of the procedure was within 30 min in all cases. Azoulay et al.^16^ reported in situ hypothermic portal perfusion under veno-venous bypass to attenuate the ischemic damage of THVE. The cold ischemic time in their series was approximately 100 min. In the current study, to minimize the duration of the occlusion, THVE was applied just before cutting of the IVC, not during hepatic parenchymal transection. These experiences show that veno-venous bypass is not mandatory, in view of the resulting hemodynamics and ischemic damage, when the duration of THVE is within 30 min.

The selection of the reconstruction method is key to success. When the major HV branch is resected, direct anastomosis usually is impossible because the position of HV was fixed on the liver. In the current study, the interposition of autologous external vein grafting was used for circumferential resection of the HV because it fit in size, as reported by other investigators.^21^ The ovarian vein graft also fits as an interposition graft for the HV (our recent experience). Regardless of the size of the defect in the HV or IVC, the crafted IMV was sufficient as a patch graft to prevent stenosis in the current study. Azoulay et al.^15^ used polytetrafluoroethylene (PTPE) vascular grafts for reconstruction of the resected major HVs and administered heparin as an anticoagulant treatment in many cases. The primary policy of the current series was to use autologous venous graft to avoid anticoagulant treatment. This may have been one of the reasons why no patient experienced hemorrhagic complications.

This study was limited by its small sample size. This could have been the reason why the long-term survival did not differ significantly between the patients with and without major vascular reconstruction (Fig. 3). Nevertheless, the survival rate for the patients with vascular reconstruction was not extremely poor. These results encourage expansion of the indication for hepatectomy using major vascular resection and reconstruction. Parenchyma-sparing hepatectomy with meticulous vascular reconstruction may become one of the standard procedures of hepatectomy for advanced CRLM.

In conclusion, the combined major vascular resection and reconstruction with hepatectomy for CRLM showed acceptable short- and long-term results. Parenchyma-sparing hepatectomy to avoid major hepatectomy is a useful option among various vascular reconstruction procedures for resection of CRLM with major vascular invasion.
